# ADHD and cognitive disengagement syndrome symptoms related to self-injurious thoughts and behaviors in early adolescents

**DOI:** 10.1007/s00787-024-02556-x

**Published:** 2024-09-05

**Authors:** Keely E. Thornton, Kelsey K. Wiggs, Jeffery N. Epstein, Leanne Tamm, Stephen P. Becker

**Affiliations:** 1 Division of Behavioral Medicine and Clinical Psychology, Cincinnati Children’s Hospital Medical Center, 3333 Burnet Avenue, MLC 10006, Cincinnati, OH 45229-3039 USA; 2 Department of Pediatrics, University of Cincinnati College of Medicine, Cincinnati, OH USA

**Keywords:** Attention-deficit/hyperactivity disorder, Comorbidity, Sluggish cognitive tempo, Self-harm, Suicide

## Abstract

The current study examined attention-deficit/hyperactivity disorder (ADHD) dimensions and cognitive disengagement syndrome (CDS) symptoms in relation to self-injurious thoughts and behaviors (SITBs) in an early adolescent sample. Participants were 341 adolescents ages 10–12 years (52.2% female; 37.8% people of color) recruited from the community. Caregivers reported on CDS and ADHD symptoms. Adolescents completed a rating scale and were administered an interview assessing SITBs. We estimated associations using logistic regression in a stepped fashion: (1) no adjustment, (2) adjustment for sex, race, family income, and psychotropic medication use, and (3) further adjustment for depressive symptoms. In this early adolescent community sample, 22.9% reported a history of suicidal ideation, 8.2% reported a history of a suicide plan, 6.2% reported a history of non-suicidal self-injury (NSSI), and 16.4% met a clinical cutoff for current suicide risk. Across most analyses using rating scale or interview methods, higher mean CDS scores were related to endorsement of suicidal ideation and planning. ADHD inattentive (IN) and hyperactive-impulsive (HI) symptoms were associated with endorsement of NSSI, and ADHD-IN symptoms were associated with thoughts of suicide and/or plan measured via questionnaire, though effects were less robust and not significant, potentially due to low base rates impacting statistical power. This study adds to a growing body of research highlighting the importance of screening for CDS symptoms among individuals with and without ADHD. More research, especially longitudinal work, is needed that examines possible differential pathways to SITBs by ADHD and CDS symptoms to advance SITB prevention, early detection, and intervention.

**Supplementary Information:**

The online version contains supplementary material available at 10.1007/s00787-024-02556-x.

Inpatient encounters for self-injurious thoughts and behaviors (SITBs) have been increasing over the past decade [1], and death by suicide has been steadily increasing among youth [2] and is now the second leading cause of death for youth aged 10–14 years old [3]. SITBs in childhood are associated with higher risk for SITBs and other adverse outcomes (e.g., mood disorders, sexual trauma) in adulthood [4, 5]. Given these concerning statistics, suicide among young people has been deemed a major public health concern [6]. There is a particular need for research focused on suicidality in childhood and early adolescence, which may enable early identification of modifiable risk factors to alter this risk trajectory [7].

Attention-deficit/hyperactivity disorder (ADHD) is one of the most prevalent psychiatric disorders in children [8], with symptoms and functional difficulties often persisting throughout adolescence and into adulthood [9, 10]. By adulthood, individuals with ADHD often experience comorbid mood disorders [11]. Given high rates of psychiatric comorbidity, coupled with well-established difficulties with emotion regulation [12], social functioning [13], low self-esteem [14], and trait impulsivity [15], researchers have hypothesized that individuals with ADHD may be at increased risk for SITBs. A recent meta-analysis [16] found support for this hypothesis, as individuals with ADHD were approximately 3.5 times more likely than those without ADHD to experience suicidal ideation, 4.5 times more likely to endorse suicidal plans, over 2 times more likely to have a suicide attempt, and over 6 times more likely to die by suicide. Importantly, this meta-analysis did not find evidence of differences in SITB risk by age bins (i.e., 6–12, 13–17, and 18+) [16]. This suggests that individuals with ADHD may be a subgroup of individuals who develop risk for SITBs early in development, underscoring the need for more research and intervention among younger samples, including in early adolescence [17].

Given that few, if any, variables reliably predict SITBs [18], and completed suicide in particular [19], it is important that research specify as much as possible which risk factors and/or subgroups of individuals are most likely to engage in SITBs. Aside from ADHD status and symptoms, there is emerging indication that cognitive disengagement syndrome (CDS; formerly termed sluggish cognitive tempo) symptoms (e.g., slowed behavior and thinking, excessive daydreaming, mental confusion) may be important for understanding SITBs in children and adolescents. CDS symptoms are distinct from, yet strongly related to, ADHD symptoms, especially inattentive symptoms, and the CDS phenotype co-occurs in 27–39% of children meeting symptom criteria for ADHD [20–23]. CDS symptoms may increase in adolescence [24] along with internalizing symptoms and SITBs [25]. CDS is associated with a number of functional difficulties—beyond what is accounted for by ADHD—that may increase the risk of SITBs including emotional dysregulation, loneliness, lowered self-esteem, and internalizing symptoms [26–28].

To date, only four studies have examined CDS in relation to SITBs. In a sample of psychiatrically hospitalized children and early adolescents (ages 8–12), Becker et al. [29] found that parent-reported CDS symptoms were uniquely associated with parent- and child-reported suicide risk (determined by composite score) above and beyond demographics, loneliness, depression, anxiety, oppositional behavior, and parental internalizing symptoms. These findings were replicated and extended in a study with college students (ages 18–29), which also found CDS symptoms to be significantly independently associated with SITBs, including lifetime suicidal ideation/attempts, past-year suicidal ideation, and suicide risk status (composite), above and beyond demographics and other psychopathologies, including ADHD, depression, and anxiety [30]. Of note, ADHD symptoms were no longer associated with SITBs when internalizing and CDS symptoms were added to the model [30]. More recently, in a sample of adolescents with and without ADHD (ages 12–14), self-reported CDS symptoms, and not ADHD-inattentive symptoms, were independently associated with higher adolescent-reported suicidal ideation (ADHD-hyperactive/impulsive symptoms were not examined) [31]. These studies provide important initial support for an association between CDS symptoms and SITBs, even when accounting for ADHD and depression.

The fourth study that has examined CDS and SITBs examined CDS in relation to non-suicidal self-injury (NSSI) in a sample of 104 adolescents (ages 12–18) with ADHD [32]. Although adolescents endorsing NSSI in the past year had higher parent-reported CDS scores than adolescents who did not endorse past-year NSSI, CDS symptoms were not independently associated with NSSI above and beyond demographics and other relevant domains. Rather, higher ADHD symptoms were independently related to past-year NSSI. This initial finding coupled with results of other studies suggests that CDS and ADHD symptoms may be differentially related to suicidal ideation/risk or NSSI, though no study has directly tested this possibility.

The purpose of the current study was to evaluate the association of ADHD symptom dimensions and CDS symptoms with numerous SITBs (e.g., total ideation, plans, attempts, NSSI) in a community-based sample of early adolescents (ages 10–12 years). Our study builds on prior research examining CDS and ADHD in relation to SITBs in important ways. First, previous studies relied on non-optimal measures of CDS and/or SITBs and the current study uses the most psychometrically-validated measure of CDS in addition to both rating scale and interview methods for assessing SITBs. Second, we examined composite SITB scores and specific domains of SITBs including suicidal ideation, suicidal plans, and NSSI. Third, studies examining ADHD and SITBs in children and adolescence have often used a composite measure of inattentive (IN) and hyperactive-impulsive (HI) symptoms (e.g., [33, 34]) though there is some indication that inattention and hyperactivity-impulsivity may be differentially associated with specific SITBs domains (e.g., [35, 33]), and additional research is needed examining separate ADHD dimensions. Based on the research reviewed above, we hypothesized that ADHD-IN, ADHD-HI, and CDS symptoms would be correlated with SITB indices, though we expected that CDS symptoms would be most strongly associated with SITBs aside from NSSI [33] when ADHD symptom dimensions and other covariates were simultaneously regressed on outcomes.

## Methods

### Participants

Participants were 341 early adolescents (ages 10–12 years) enrolled in a broader study of attention problems and mental health. Participant and caregiver/family characteristics are summarized in Table 1. There were approximately an equal number of female and male adolescents, with slightly over one-third of participants being people of color. Approximately half of the sample met diagnostic criteria for ADHD based on the K-SADS interview [37] conducted with the adolescent’s caregiver (primarily biological mothers).

**Table 1:** Sample characteristics

	Total Sample (*N* = 341)
	*M* ± *SD*
Age	10.90 ± 0.80
	*n* (%)
Female	178 (52.2)
Male	163 (47.8)
Race	
American Indian/Alaskan	1 (0.3)
Asian	8 (2.3)
Black	72 (21.1)
Multiracial	48 (14.1)
White	212 (62.2)
Hispanic/Latiné	32 (9.4)
Relationship to Child	
Biological Mother	293 (85.9)
Biological Father	26 (7.6)
Stepmother	2 (0.6)
Adoptive Mother	14 (4.1)
Adoptive Father	1 (0.3)
Foster Mother	1 (0.3)
Grandmother	3 (0.9)
Grandfather	1 (0.3)
Household Income ^a^	
Under 20,000	15 (4.5)
20,001–40,000	37 (11.1)
40,001–60,000	34 (10.2)
60,001–80,000	38 (11.4)
80,001-100,000	34 (10.2)
100,001-120,000	45 (13.5)
Over 120,000	130 (39)
Medication	
ADHD (any)	83 (24.3)
Methylphenidate	50 (14.7)
Amphetamine^b^	26 (7.6)
Non-stimulant^c^	19 (5.6)
Other Psychiatric (any)	
Antidepressant/antianxiety	24 (7)
Psychiatric diagnoses^b^	
ADHD - (Caregiver-report)	166 (48.7)
ADHD-IN	105 (30.8)
ADHD-HI	5 (1.5)
ADHD-Combined	56 (16.4)
ADHD - Adolescent-report	85 (25.1)
ADHD-IN	42 (12.3)
ADHD-HI	6 (1.8)
ADHD-Combined	37 (10.9)
Any externalizing (ODD)^c, d^- Caregiver-report	24 (7)
Any anxiety - Caregiver-report	52 (15.2)
Any anxiety - Adolescent-report	39 (11.4)
Any depression - Caregiver-report	5 (1.5)
Any depression - Adolescent-report	8 (2.3)

*Note*^a^8 caregivers did not provide family income

^b^ADHD=attention-deficit/hyperactivity disorder. ADHD-IN = ADHD, inattentive presentation. ADHD-HI = ADHD, hyperactive/impulsive presentation. ODD = oppositional defiant disorder. Anxiety disorders = presence of generalized anxiety disorder, social phobia, panic disorder and/or posttraumatic stress disorder (PTSD). Any depression = presence of major depression or dysthymia

^c^No participants met criteria for conduct disorder

^d^Presence of comorbid mental health diagnosis based on caregiver or adolescent report (only caregivers were administered ODD and PTSD modules) during the diagnostic interview.

### Procedures

All procedures were approved by the Cincinnati Children’s Hospital Medical Center Institutional Review Board. Caregivers and adolescents provided informed consent and assent, respectively. Adolescents and their caregivers were recruited for a prospective longitudinal study on CDS from a variety of sources, including media advertisements (e.g., television, Facebook, Instagram, Nextdoor), community flyers, e-mail distribution within a Midwestern children’s hospital, and letters to school/pediatrician partners. To assure a range of CDS symptomatology, several versions of advertising materials were generated (e.g., some specifying daydreaming, mental confusion, and slowed behavior/thinking, and some not targeting any attentional problems). Longitudinal data collection is ongoing; thus, only baseline data are used in the present study.

Interested caregivers completed a brief REDCap eligibility survey that included initial inclusion criteria. Families meeting initial inclusion criteria were scheduled for an in-person research visit, during which remaining eligibility criteria were assessed. In addition to being ages 10–12 years, inclusion criteria included a standardized score ≥ 80 for overall intelligence on the Peabody Picture Vocabulary Test, 5th Edition [38], willingness to discontinue stimulant medications for ADHD 24 h prior to their research visit, and sufficient English language ability to complete the measures. Adolescents were excluded if the caregiver reported a previous diagnosis of autism spectrum disorder, bipolar disorder, or psychosis, or a significant visual, hearing or speech impairment precluding their ability to complete the measures. For additional details, see Becker et al. [39].

### Measures

#### Demographic characteristics and medication use

Caregivers completed a demographic form to gather the information reported in Table 1. Medication use and psychosocial treatment were assessed with an adaptation of the Service Assessment for Children and Adolescents (SACA) [40].

##### Kiddie schedule for affective disorders and schizophrenia for school-age children (K-SADS)

The K-SADS [37] is a semi-structured diagnostic interview based on the DSM-5 with good reliability and validity [41]. In the present study, interviews conducted with the adolescent’s caregiver were used to assess ADHD, oppositional defiant disorder (ODD), and conduct disorder (CD), whereas interviews conducted with the adolescent were used to assess generalized anxiety disorder, social phobia, panic disorder, post-traumatic stress disorder, major depression, and persistent depressive disorder modules. The K-SADS was administered by trained graduate students, post-doctoral fellows, or clinical psychologists.

##### Child and adolescent behavior inventory (CABI)

Caregivers completed the 18-item ADHD (i.e., 9 ADHD-IN items, 9 ADHD-HI items) module and 15-item CDS module from the CABI [42]. Symptoms were rated with 6-point anchors for the past month (0 = *almost never [never or about once per month]*, 1 = *seldom [about once per week]*, 2 = *sometimes [several times per week]*, 3 = *often [about once per day]*, 4 = *very often [several times per day]*, and 5 = *almost always [many times per day]*). Earlier studies support the factor structure, reliability (internal consistency, test-retest, and interrater) and validity of CABI scale scores from the United States [21, 43, 44]. Other studies from Iran, South Korea, Spain, Turkey, and the United States also support the psychometric properties of CABI scale scores [45–50]. Studies also support using the CABI as a unidimensional measure of CDS symptoms [51, 52]. For a review of the CABI CDS measure, see Becker [53]. In the present study, mean ADHD-IN, ADHD-HI, and CDS symptom scores were the primary predictors of interest.

##### Depressive symptom index-suicidality scale (DSI-SS)

Adolescent’s self-reported intensity and frequency of suicidal ideation and impulses were assessed using the DSI-SS [54]. The DSI-SS consists of 4 items: thoughts of killing self, thoughts of suicide and/or plan, having little or no control over suicidal thoughts, and having impulses to kill self. Each item is scored from 0 to 3 (e.g., “I do not have thoughts of killing myself”=0, “Sometimes I have thoughts of killing myself”=1, “Most of the time I have thoughts of killing myself”=2, “I always have thoughts of killing myself”=3), with a total composite score ranging from 0 to 12. For each individual item and the composite score, higher scores indicate a greater frequency and severity of suicidal ideation. The DSI-SS has been shown to have strong psychometric properties [55]. In the present study, we used a cutoff score of 1 to categorize suicide risk based on prior work that has normed this cutoff using the DSI-SS in outpatient settings [56], and we also examined associations with dichotomized individual items where scores > 0 were considered elevated.

##### Self-injurious thoughts and behaviors interview (SITBI)

History of lifetime suicidal ideation, suicide plan, aborted attempts, suicide attempts, and NSSI were assessed using the SITBI [57], a structured interview that assesses the presence, frequency, and characteristics of a wide range of SITBs. The SITBI has demonstrated strong interrater reliability, test-retest reliability, and intraclass correlation over a six-month period, as well as concurrent validity with other measures of suicidal ideation [57]. As in previous research [58–60], adolescent’s responses to whether they had ever had thoughts of killing themself (i.e., suicidal ideation; 0 = *never*, 1 = *once or twice*, 2 = *a few times*, 3 = *many times*), ever thought or a way or method to kill themself (i.e., suicide plan; 0 = *never*, 1 = *once or twice*, 2 = *a few times*, 3 = *many times*) were dichotomized (i.e., scores > 0) and included as outcome variables. We also examined reports of history of ever purposely hurt themself without wanting to die (i.e., NSSI; 0 = *no*, 1 = *yes*). Very few participants endorsed aborted attempts (*n* = 5 of 334 participants who answered this item, 1.50%) or suicide attempts (*n* = 2 of 335 participants, 0.60%), and so these variables were not examined in the current study.

##### Behavioral assessment system for children (BASC-3) self-report of personality (SRP)

Adolescents completed the self-report BASC-3 SRP [61], a multidimensional measure with items rated on a four-point scale of frequency (0 = *never*, 3 *= almost always*). In the current study, the depression scale was used as a covariate (none of the BASC-3 SRP depression scale items directly assess SITBs).

#### Data analytic approach

We used SPSS version 29 for descriptive analyses and SAS version 9 for modeling. We first estimated bivariate associations between all study variables using Pearson correlations (including point-biserial correlations for correlations between dichotomous and continuous variables) and chi-square tests. Next, we estimated the likelihood (i.e., odds) of SITB indices associated with ADHD-IN, ADHD-HI, and CDS symptoms using logistic regression. We estimated the likelihood of score elevations in our outcome variables (i.e., dichotomized) in order to maximize power given the extremely low frequencies across ratings above 0. Initial estimates were unadjusted for any influence of covariates. Next, relevant participant characteristics including sex, race, family income, and medication status were included as covariates. Finally, we added mean adolescent-reported depressive symptoms as a covariate to provide the most stringent test of associations, independent of possible differences in depressive symptoms. Overall, we had low levels of missing data (total of 10 participants had missing on at least one measure used in this study, including 1 participant without SITBI data and 1 separate participant without DSI-SS data; see Table 1), analyses excluded individuals with missing information on an analysis by analysis basis.

## Results

### Descriptive analyses

On the DSI-SS, 16.4% (*n* = 56) met the cutoff for suicide risk on the DSI-SS. For individual items, 40 (11.7%) endorsed thoughts of killing themselves, 28 (8.2%) endorsed the suicide plan item, 26 (10.6%) endorsed at least some lack of control over suicidal thoughts, and 25 (7.3%) endorsed impulses to kill themselves. On the SITBI, 22.9% (*n* = 78) of participants reported a history of suicidal ideation, 8.2% (*n* = 28) reported a history of a suicide plan, and 6.2% (*n* = 21) reported a history of NSSI.

Correlations among study variables are presented in Table 2. Of note, higher mean CDS symptoms were weakly correlated with all SITB indices (*r*s = 0.14-0.22, *p*s < 0.05) with the exception of NSSI (*p* > .05). Higher mean ADHD-IN symptoms were weakly correlated with endorsement of thoughts of killing self and thoughts of suicide and/or plan on the DSI-SS, as well as NSSI (*r*s = 0.11-0.13; *p*s < 0.05) but not with other SITB indices (*p*s > 0.05). ADHD-HI symptoms were not significantly correlated with any SITB outcome (all *p*s > 0.05).

**Table 2:** Bivariate correlations and chi square statistics among primary study measures

Variable	1	2	3	4	5	6	7	8	9
1. Female	--	--	--	--	--	− 0.19	− 0.18**	− 0.08	− 0.02
2. Race (non-White)	--	--	--	--	--	− 0.01	− 0.00	− 0.11*	0.02
3. Hispanic/Latiné	--	--	--	--	--	− 0.11*	− 0.09	− 0.04	0.02
4. Income (below median)		--	--	--	--	− 0.00	− 0.07	− 0.12*	0.06
5. Any Medication	--	--	--	--	--	0.46**	0.47**	0.25**	0.27**
6. ADHD-IN Symptoms	--	--	--	--	--	--	0.69**	0.70**	0.20**
7. ADHD-HI Symptoms	--	--	--	--	--	--	--	0.45**	0.10
8. CDS Symptoms	--	--	--	--	--	--	--	--	0.20**
9. BASC Depressive Symptoms	--	--	--	--	--	--	--	--	--
10. DSI-SS Composite	0.01	1.28	1.87	4.76*	2.36	0.08	0.03	0.17**	0.35**
11. DSI-SS Item 1: Thoughts of killing self	0.14	0.99	0.52	2.30	3.51	0.13*	0.04	0.22*	0.36**
12. DSI-SS Item 2: Thoughts of suicide and/or plan	0.89	0.06	2.58	0.66	5.33	0.13*	0.01	0.19**	0.26**
13. DSI-SS Item 3: Lack of control of suicidal thoughts	0.61	0.25	2.51	3.34	5.74*	0.10	0.01	0.14**	0.30**
14. DSI-SS Item 4: Impulses to kill self	0.16	1.19	3.58	3.32	0.00	0.04	0.01	0.14**	0.19**
15. SITBI: Suicidal Ideation	0.49	0.86	1.69	0.74	1.40	0.10	0.04	0.17**	0.27**
16. SITBI: Suicidal Plans	0.02	0.03	5.20*	0.07	2.10	0.10	0.04	0.20**	0.23**
17. SITBI: NSSI	3.37	0.84	2.66	2.85	2.71	0.11*	0.11	0.07	0.09

*Note* Continuous variable correlations calculated with standard Pearson r. Continuous and dichotomous correlations calculated with Pearson bi-serial r. Dichotomous variable statistics calculated with Chi Square tests. *p < .05. **p < .01

### Logistic regression models

We present multivariate logistic regression results in Tables 3 and 4.

**Table 3:** Point estimates for multivariate associations between CDS, ADHD-IN, and ADHD-HI symptoms and DSI-SS outcomes

	CDS	ADHD-IN	ADHD-HI
	OR (95% CI)	OR (95% CI)	OR (95% CI)
Total DSI-SS Composite			
Unadjusted	**1.74 (1.16–2.61)**	0.90 (0.64–1.27)	0.97 (0.71–1.32)
Adjusted 1^a^	**1.67 (1.10–2.58)**	0.90 (0.62–1.31)	0.84 (0.60–1.16)
Adjusted 2^b^	1.50 (0.95–2.36)	0.87 (0.59–1.30)	0.88 (0.62–1.26)
DSI-SS Item 1: Thoughts of killing self			
Unadjusted	**1.90 (1.22–2.95)**	1.05 (0.71–1.55)	0.87 (0.62–1.21)
Adjusted 1^a^	**2.04 (1.29–3.23)**	0.98 (0.64–1.48)	0.81 (0.57–1.15)
Adjusted 2^b^	**1.87 (1.14–3.08)**	0.96 (0.61–1.49)	0.89 (0.61–1.30)
DSI-SS Item 2: Thoughts of suicide and/or plan			
Unadjusted	**1.71 (1.04–2.82)**	1.30 (0.84–2.01)	0.71 (0.48–1.06)
Adjusted 1^a^	**1.77 (1.06–2.96)**	1.20 (0.75–1.92)	**0.66 (0.43–0.998)**
Adjusted 2^b^	1.62 (0.96–2.74)	1.20 (0.84–1.95)	0.71 (0.46–1.09)
DSI-SS Item 3: Lack of control of suicidal thoughts			
Unadjusted	1.46 (0.93–2.31)	1.16 (0.78–1.73)	0.81 (0.56–1.15)
Adjusted 1^a^	1.55 (0.97–2.50)	1.06 (0.69–1.62)	0.73 (0.50–1.07)
Adjusted 2^b^	1.41 (0.86–2.31)	1.05 (0.67–1.64)	0.80 (0.54–1.19)
DSI-SS Item 4: Impulses to kill self			
Unadjusted	**2.17 (1.25–3.78)**	0.76 (0.45–1.26)	0.96 (0.63–1.49)
Adjusted 1^a^	**2.28 (1.29–4.04)**	0.75 (0.44–1.27)	0.93 (0.60–1.43)
Adjusted 2^b^	**2.08 (1.16–3.72)**	0.73 (0.42–1.26)	0.99 (0.63–1.56)

*Note* CDS = cognitive disengagement syndrome. ADHD = attention-deficit/hyperactivity disorder. IN = inattentive. HI = hyperactive-impulsive. DSI-SS = Depressive Symptom Index-Suicidality Scale. OR = odds ratio. CI = confidence interval

^a^Adjusted for sex, race, family income, and medication status

^b^Adjusted for child and family factors in addition to depressive symptoms

**Table 4:** Point estimates for multivariate associations between CDS, ADHD-IN, and ADHD-HI symptoms and SITBI outcomes

	CDS	ADHD-IN	ADHD-HI
	OR (95% CI)	OR (95% CI)	OR (95% CI)
SITBI: Suicidal Ideation			
Unadjusted	**1.57 (1.09–2.24)**	1.00 (0.74–1.35)	0.93 (0.71–1.21)
Adjusted 1^a^	**1.66 (1.15–2.41)**	0.94 (0.69–1.29)	0.88 (0.67–1.16)
Adjusted 2^b^	**1.56 (1.06–2.28)**	0.92 (0.66–1.27)	0.93 (0.70–1.24)
SITBI: Suicidal Plans			
Unadjusted	**2.12 (1.27–3.53)**	0.93 (0.58–1.48)	0.91 (0.61–1.35)
Adjusted 1^a^	**2.23 (1.32–3.78)**	0.86 (0.53–1.41)	0.86 (0.58–1.29)
Adjusted 2^b^	**2.05 (1.19–3.52)**	0.84 (0.51–1.40)	0.94 (0.62–1.43)
SITBI: NSSI			
Unadjusted	0.98 (0.54–1.77)	1.24 (0.75–2.05)	1.17 (0.75–1.81)
Adjusted 1^a^	0.95 (0.52–1.72)	1.26 (0.75–2.12)	1.21 (0.75–1.93)
Adjusted 2^b^	0.91 (0.50–1.66)	1.23 (0.73–2.08)	1.27 (0.79–2.05)

*Note* CDS = cognitive disengagement syndrome. ADHD = attention-deficit/hyperactivity disorder. IN = inattentive. HI = hyperactive-impulsive. SITBI = Self-Injurious Thoughts and Behavior Interview. NSSI = non-suicidal self-injury. OR = odds ratio. CI = confidence interval

^a^Adjusted for sex, race, family income, and medication status

^b^Adjusted for child and family factors in addition to depressive symptoms

#### DSI-SS

In unadjusted models (i.e., those that only included our 3 predictors of interest), CDS symptoms were the only significant predictor of our composite DSI-SS variable, such that the odds of elevations on the DSI-SS were increased by 74% for each 1-point increase in CDS mean score (95% CI = 1.16–2.61). This association was attenuated, though still significant after adjustment for participant characteristics (OR = 1.67, 95% CI = 1.10–2.58). However, although we still observed a 50% higher odds of DSI-SS elevations for each 1-point increase in CDS symptoms following further adjustment for depressive symptoms, this estimate could not be distinguished from the null hypothesis (Table 3). Neither ADHD-IN nor ADHD-HI symptoms were related to higher odds of elevated DSI-SS composite scores.

**Fig. 1:**
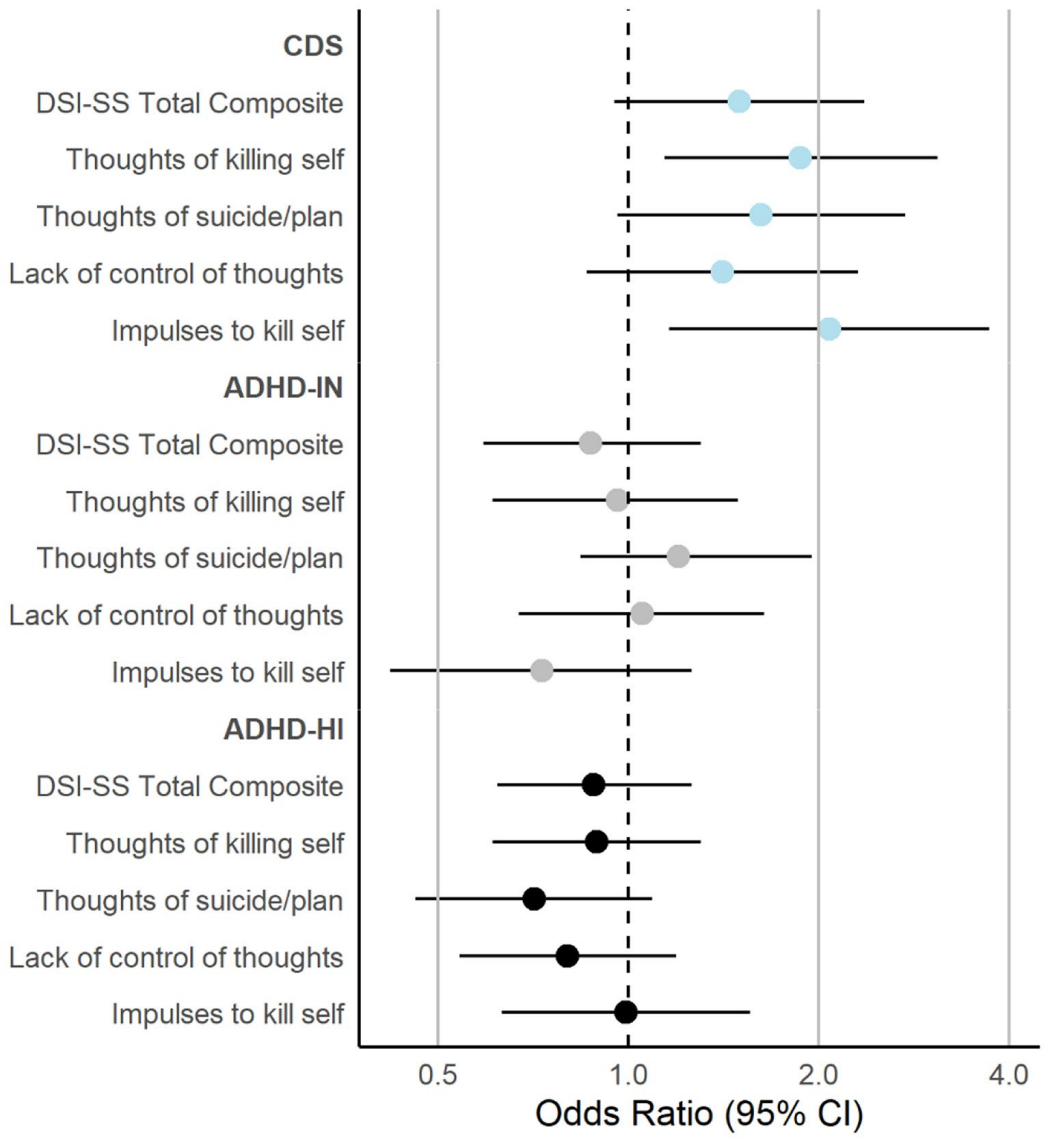
Logistic analyses examining the unique effects of ADHD-IN, ADHD-HI, and CDS symptoms in relation to DSI-SS indices associated in fully adjusted models. Note: All models included CDS, ADHD-IN, and ADHD-HI symptoms dimensions simultaneously, in addition to including sex, race, family income, medication status, and depressive symptoms as covariates

In examining individual items on the DSI-SS, we observed a similar pattern for most DSI-SS items. The odds of endorsement of suicidal thoughts or plans increased by 71% with each 1-point increase in mean CDS symptoms in unadjusted models (95% CI = 1.04–2.82), which increased to 77% after adjustment for participant characteristics (OR = 1.06–2.96). Though adjustment for depressive symptoms again attenuated this association to the point of non-significance (OR = 1.62, 95% CI = 0.96–2.74). Higher mean scores in CDS symptoms, however, were related to elevations in thoughts of killing self (fully adjusted OR = 1.87, 95% CI = 1.22–2.95) and impulses to kill self (fully adjusted OR = 2.08, 95% CI = 1.16–3.72) across adjustment in all models. Although higher CDS symptom scores were associated with higher odds of a lack of control over suicidal thoughts, the association was not able to be differentiated from the null hypothesis in any model (fully adjusted OR = 1.41, 95% CI = 0.86–2.31. Finally, although neither ADHD-IN nor ADHD-HI symptoms were not significantly related to higher odds any DSI-SS item, higher ADHD-IN symptoms were still associated with non-significantly higher odds of suicidal thoughts and plans (fully adjusted OR = 1.20, 95% CI = 0.84–1.95; Table 3). Figure 1 provides a summary of CDS, ADHD-IN, and ADHD-HI symptoms in relation to DSI-SS variables in the fully adjusted model (figures for non-fully adjusted models are presented in Supplemental Materials).

#### SITBI

**Fig. 2:**
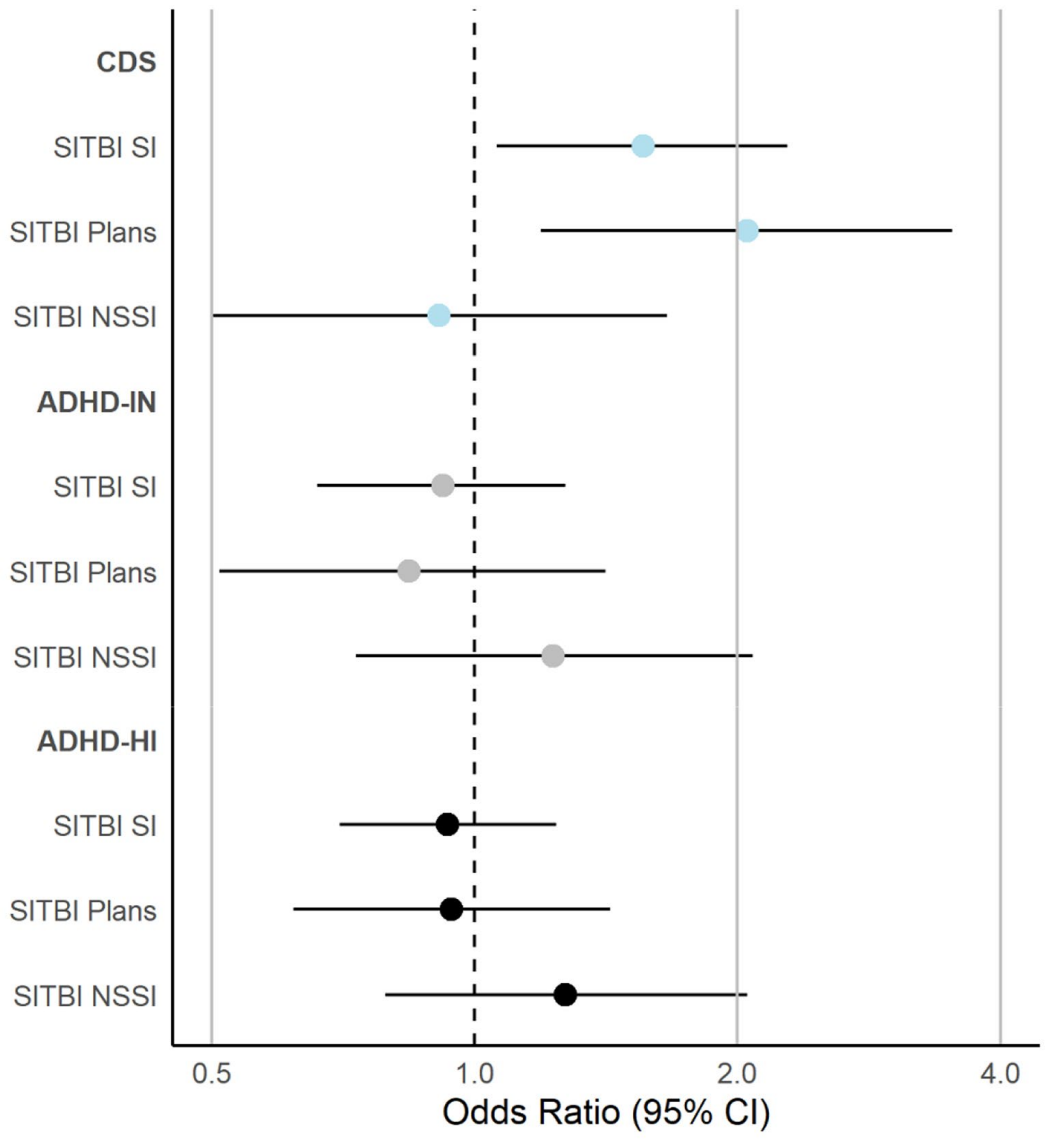
Logistic analyses examining the unique effects of ADHD-IN, ADHD-HI, and CDS symptoms in relation to SITBI indices associated in fully adjusted models. Note: All models included CDS, ADHD-IN, and ADHD-HI symptoms dimensions simultaneously, in addition to including sex, race, family income, medication status, and depressive symptoms as covariates

Similar to our findings measuring suicidal thoughts and plans via questionnaire, our findings via interview examining suicidal thoughts (fully adjusted OR = 1.56, 95% CI = 1.06–2.28) and plans (fully adjusted OR = 2.05, 95% CI = 1.19–3.52) suggested that these outcomes were only significantly associated with CDS symptoms, but not ADHD-IN or ADHD-HI symptoms, across models. Finally, in contrast to most other outcomes, CDS symptoms were not related to NSSI (OR range = 0.98 − 0.91 across adjustment). Instead, higher mean scores of both ADHD-IN (fully adjusted OR = 1.23, 95% CI = 0.73–2.08) and ADHD-HI symptoms (fully adjusted OR = 1.27, 95% CI = 0.79–2.05) were related. However, model estimates were not distinguishable from the null hypothesis (Table 4). Figure 2 provides a summary of findings for CDS, ADHD-IN, and ADHD-HI symptoms in relation to SITBI variables in the fully adjusted model (figures for non-fully adjusted models are presented in Supplemental Materials).

## Discussion

This study builds on prior literature examining CDS, ADHD-IN, and ADHD-HI symptoms in relation to SITBs in a community-based sample of early adolescents. Although previous literature has demonstrated an association between CDS and SITBs, no study has included multi-informant rating scales and interviews for SITBs assessing many separate thoughts and behaviors along the SITB continuum (including but not limited to plans, methods, and NSSI). As hypothesized, CDS symptoms, but not ADHD-IN or ADHD-HI symptoms, were most strongly and robustly associated with early adolescent endorsement SITBs generally across our rating scale and interview, even when controlling for participant characteristics (i.e., sex, household income, race, and medication use) and adolescent-reported depressive symptoms. The only exception to this was for NSSI, though associations with ADHD-IN and ADHD-HI symptoms were not significant.

Across both rating scale and interview methods, higher mean CDS symptoms were related to endorsement of suicidal ideation across all adjustments. We also observed evidence that early adolescents with higher CDS symptoms may engage in suicidal planning more often than others, though we only observed a significant association from our questionnaire prior to adjustment for depressive symptoms (and ADHD-IN symptoms were also more weakly associated with endorsement on our questionnaire, though associations were not significant). Finally, we observed evidence that CDS symptoms may be uniquely related to adolescents’ impulses to kill themselves across adjustment for all covariates.

There are multiple possible reasons that rates of suicidal ideation may be uniquely related to CDS symptoms. One explanation may be the relevance of CDS to the interpersonal theory of suicide [62], which posits that thwarted belongingness (feeling socially disconnected and without support) and perceived burdensomeness (self-hatred and beliefs that one is a liability to others) contribute to suicidal thoughts/desire. The added presence of acquired capability for suicide (lowered fear of death and elevated physical pain tolerance) is when near-lethal and lethal attempts are most likely to occur [62]. In considering thwarted belongingness, social isolation is one of the strongest and most reliable predictors of SITBs [62], and studies have consistently found CDS to be uniquely associated with social withdrawal and isolation [52, 63–66]. In addition, low self-esteem is a core component of perceived burdensomeness [62] and is also significantly associated with CDS symptoms [67, 68] and ADHD [69, 70]. Considered together, it is plausible that adolescents with ADHD and co-occurring CDS symptoms experience social isolation and lowered self-esteem which make them more likely to also experience suicidal ideation and desire. However, we should also note that our findings regarding weaker and non-significant associations between ADHD-IN symptoms and thoughts of suicide and/or plan may warrant further investigation considering the low base rate and thus low power we had in estimating associations.

In contrast to our findings for suicidal ideation and suicidal planning, we did not find CDS to be associated with early adolescent endorsement of engaging in NSSI. Rather, ADHD-IN and ADHD-HI symptoms were both related to almost a 30% increase in odds for each 1-point increase in mean symptom scores, though these elevations were not significant. However, low power may have again limited our ability to observe meaningful associations. Our findings are in line with another recent study which found higher ADHD symptoms, but not CDS symptoms, to be associated with past-year NSSI in adolescents with ADHD [32].

If meaningful differences do exist among CDS and ADHD symptoms regarding NSSI and other SITB indices, findings may suggest differential pathways into SITBs that warrant further investigation with larger or higher-powered samples. More specifically, our findings might provide preliminary evidence that early adolescents with higher CDS symptoms are more likely to engage in suicidal *ideation and plans*, whereas early adolescents with higher ADHD symptoms are more likely to engage in non-suicidal (and perhaps even suicidal) self-harm *behaviors*. When CDS and ADHD-IN are considered simultaneously, CDS symptoms are often negatively associated with hyperactive-impulsive symptoms [71], and it is possible that the presence of hyperactive and impulsive behaviors distinctly contribute to behaviors such as NSSI. Further, ADHD and CDS are hypothesized to arise from different neural substrates [72, 73], and trait-impulsivity underlying externalizing psychopathology (including ADHD) may be important for understanding youth who are more likely to engage in self-harm behaviors. More research is needed that examines (1) possible differential associations between ADHD and CDS symptom dimensions and SITB indices, (2) CDS in relation to the proximal components of the interpersonal model of suicide, and (3) developmental trajectories that may be involved in the progression along the SITB continuum.

Further, if replicated in independent samples, our findings may have important implications for assessment, including incorporation of CDS in ADHD-focused assessments as well as the importance of assessing for a range of SITB indices. In addition, although CDS is as more trait-like than state-like [74, 75], symptoms may nevertheless fluctuate for a multitude of reasons (e.g., poor/insufficient sleep, peer difficulties; see [76, 77]) and be important to monitor as part of ongoing mental health screening or treatment.

### Strengths, limitations, and future directions

Strengths of the study include using a psychometrically-valid measure of CDS, employing multiple methods for assessing multiple domains of SITBIs, recruiting a diverse community sample, and recruiting outside the confines of ADHD specifically. However, there are several limitations that are important to note, which should influence further research. First, as already stated, there was a low base rate of NSSI and more serious SITB indices (e.g., plans, attempts) in our early adolescent sample, limiting statistical power. Second, because recruitment targeted youth to reflect the full range of CDS symptomology, the sample also reflected a larger proportion of adolescents with predominately ADHD-IN, such that findings regarding ADHD-HI symptoms may be limited. Third, although this study was more diverse both in race and sex than previous studies, the sample was still predominantly White (60%) with a relatively high socioeconomic status, which may limit the generalizability of our findings. Fourth, future research would benefit from longitudinal data to investigate possible differential developmental trajectories discussed above, including the possible mechanisms linking CDS and ADHD to SITBs including direct measurement of constructs within the interpersonal model of suicide.

In conclusion, the current study examined the associations between CDS, ADHD-IN, and ADHD-HI symptoms and multiple SITB indices among early adolescents, finding that CDS symptoms were most strongly associated with suicidal ideation and suicidal plans, even after adjustment for participant characteristics and depressive symptoms. Additional research is needed to measure associations longitudinally and examine potential mechanisms between CDS, ADHD symptom dimensions, and SITBs throughout adolescence.

## Electronic supplementary material

Below is the link to the electronic supplementary material.

Supplementary Material 1

## Data Availability

Data is available from the corresponding author following execution of a data use agreement.
