# Supplementary material for: ADHD and cognitive disengagement syndrome symptoms related to self-injurious thoughts and behaviors in early adolescents
Source: Eur Child Adolesc Psychiatry. 2024 Sep 5;34(3):1195–206. doi: 10.1007/s00787-024-02556-x (PMC11909033; doi:10.1007/s00787-024-02556-x)

**SUPPLEMENTARY MATERIAL**

Figure S1

*Logistic analyses examining the unique effects of ADHD-IN, ADHD-HI, and CDS symptoms in relation to DSI-SS indices associated in unadjusted models*


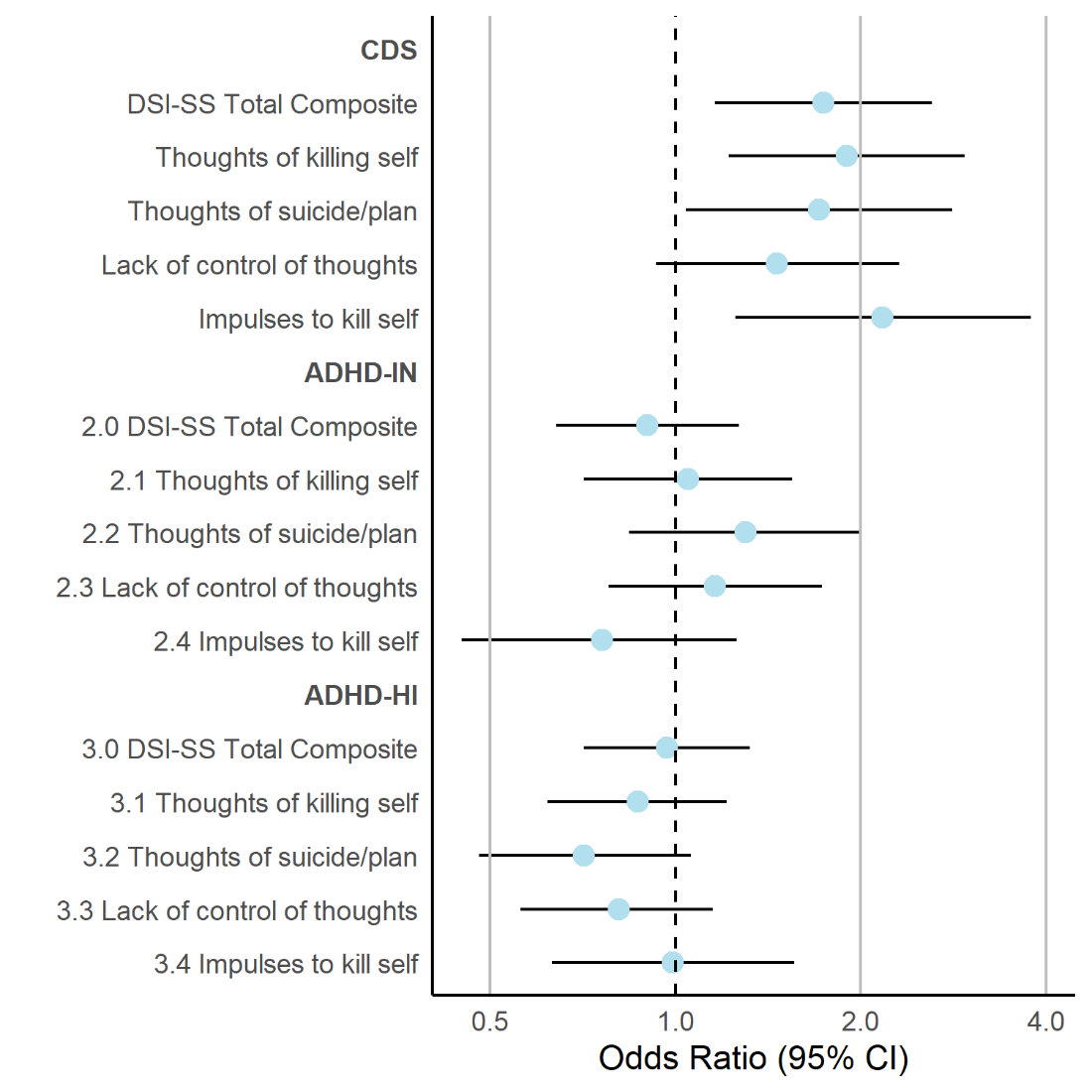

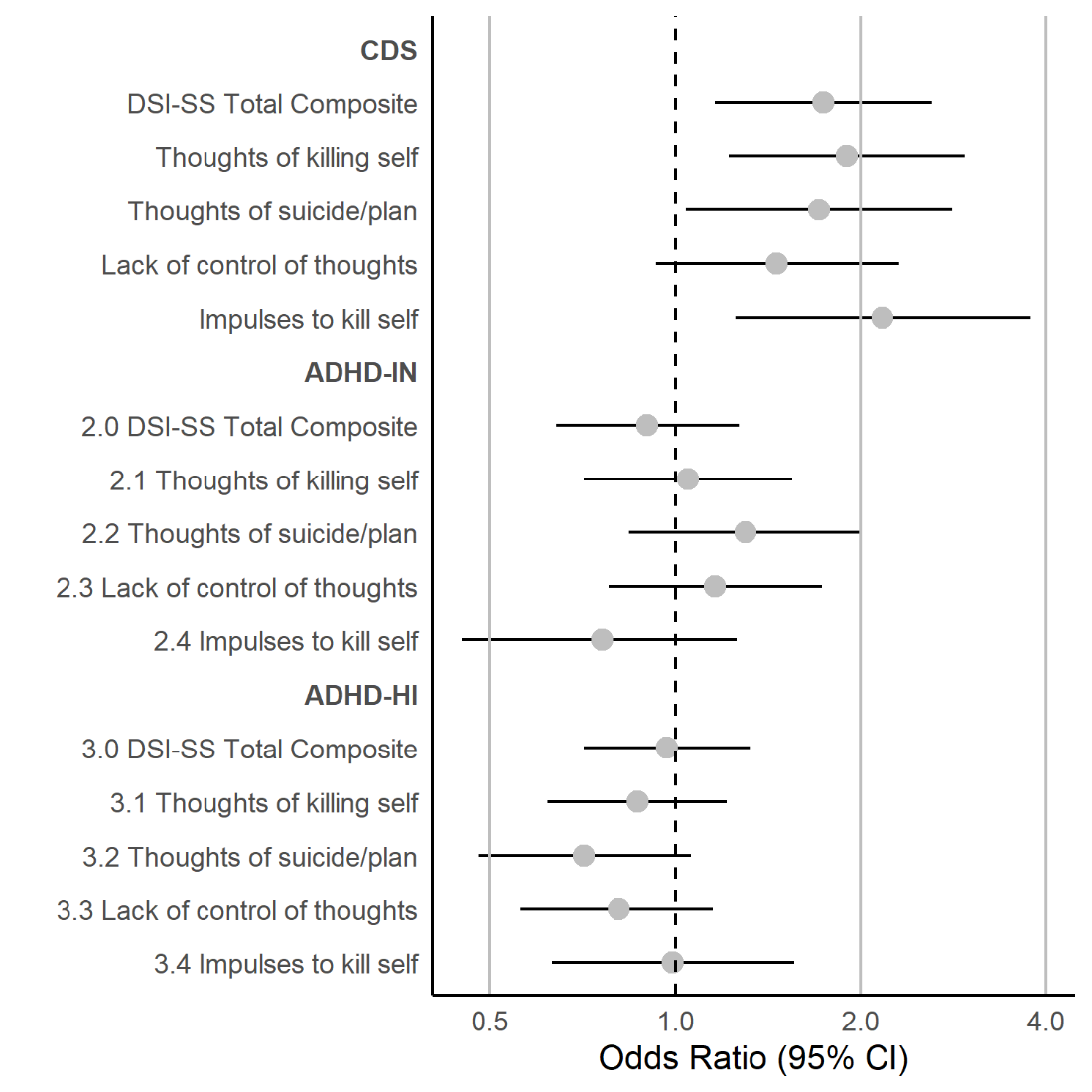

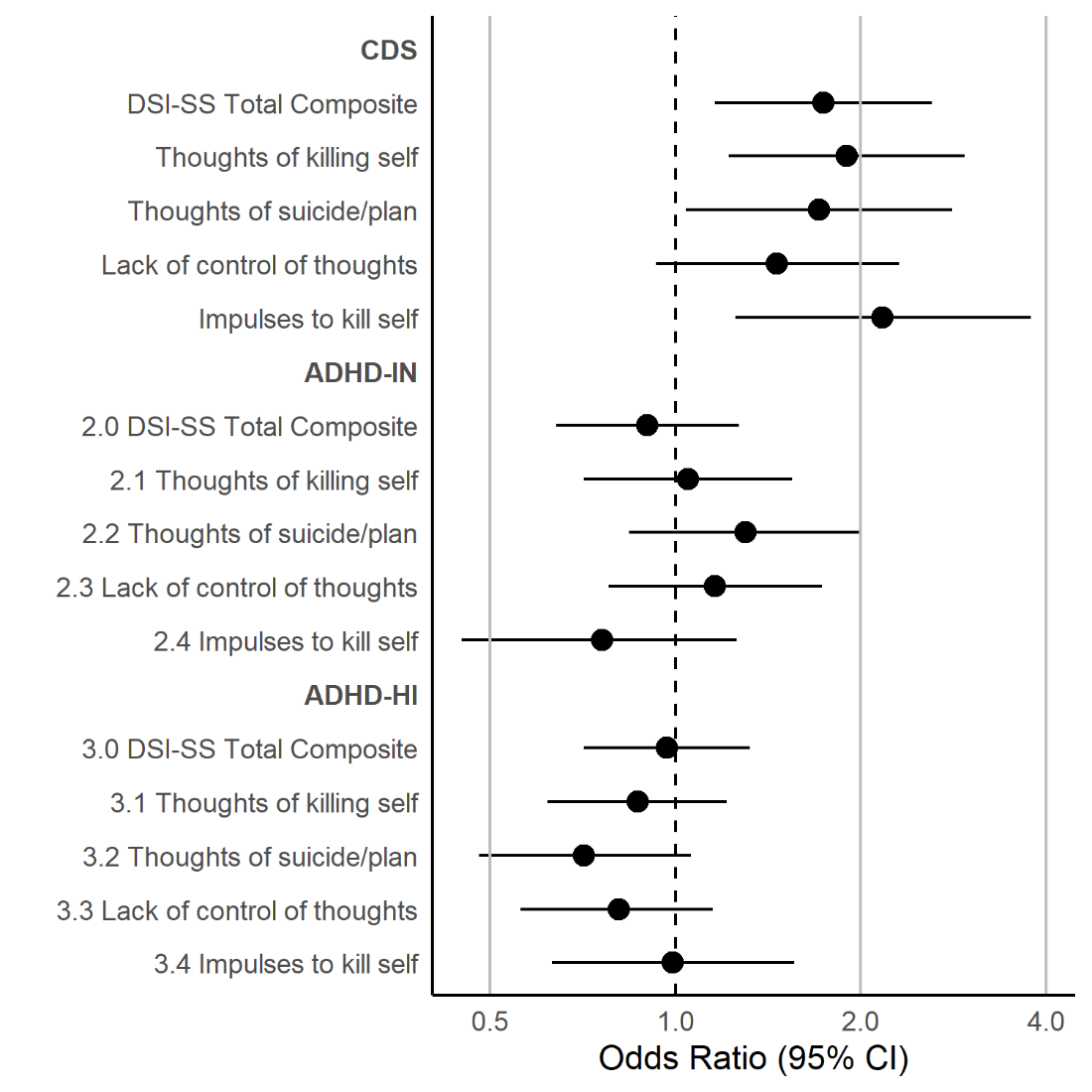


Figure S2

*Logistic analyses examining the unique effects of ADHD-IN, ADHD-HI, and CDS symptoms in relation to DSI-SS indices associated in models adjusted for demographic characteristics*


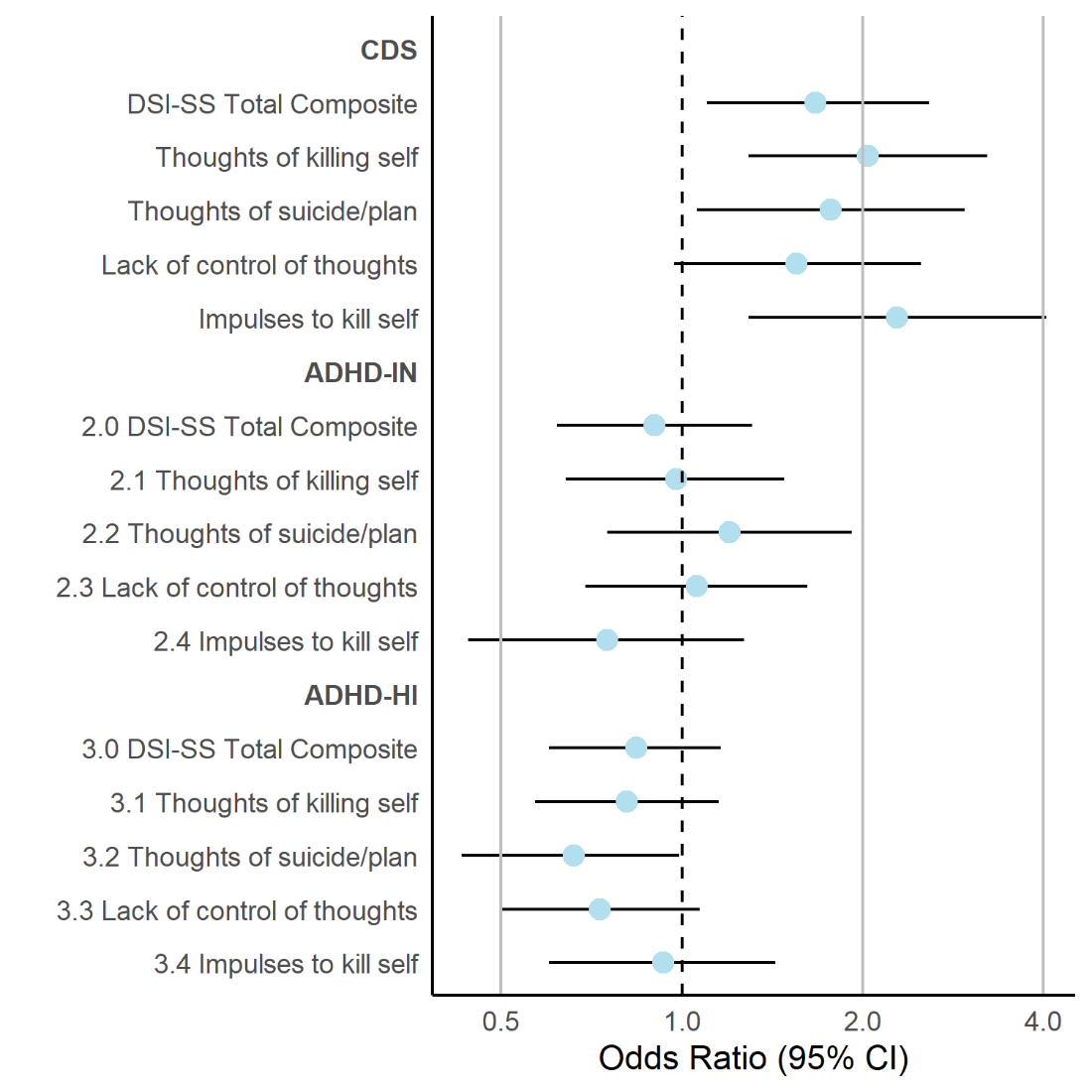

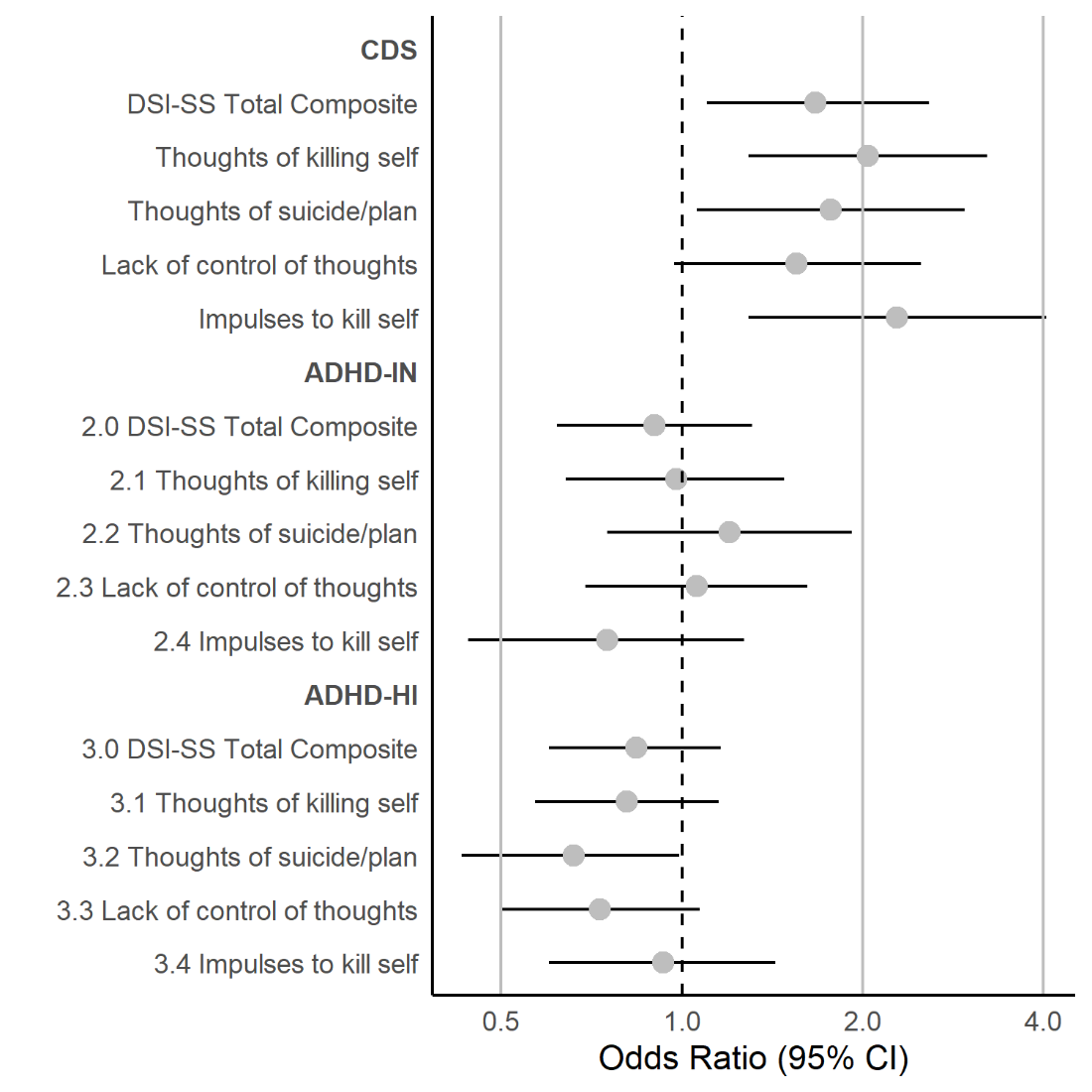

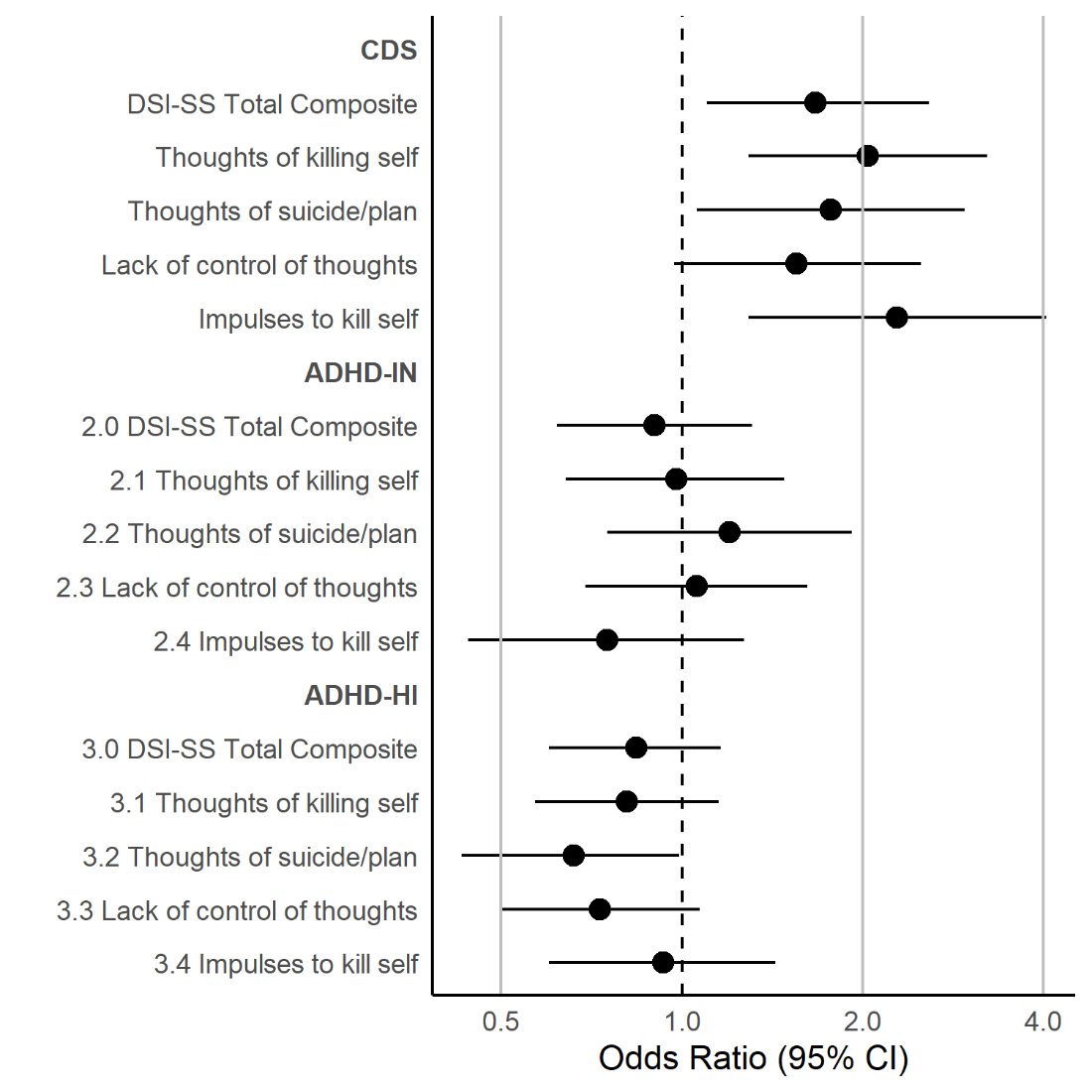


Figure S3

*Logistic analyses examining the unique effects of ADHD-IN, ADHD-HI, and CDS symptoms in relation to SITBI indices associated in unadjusted models*


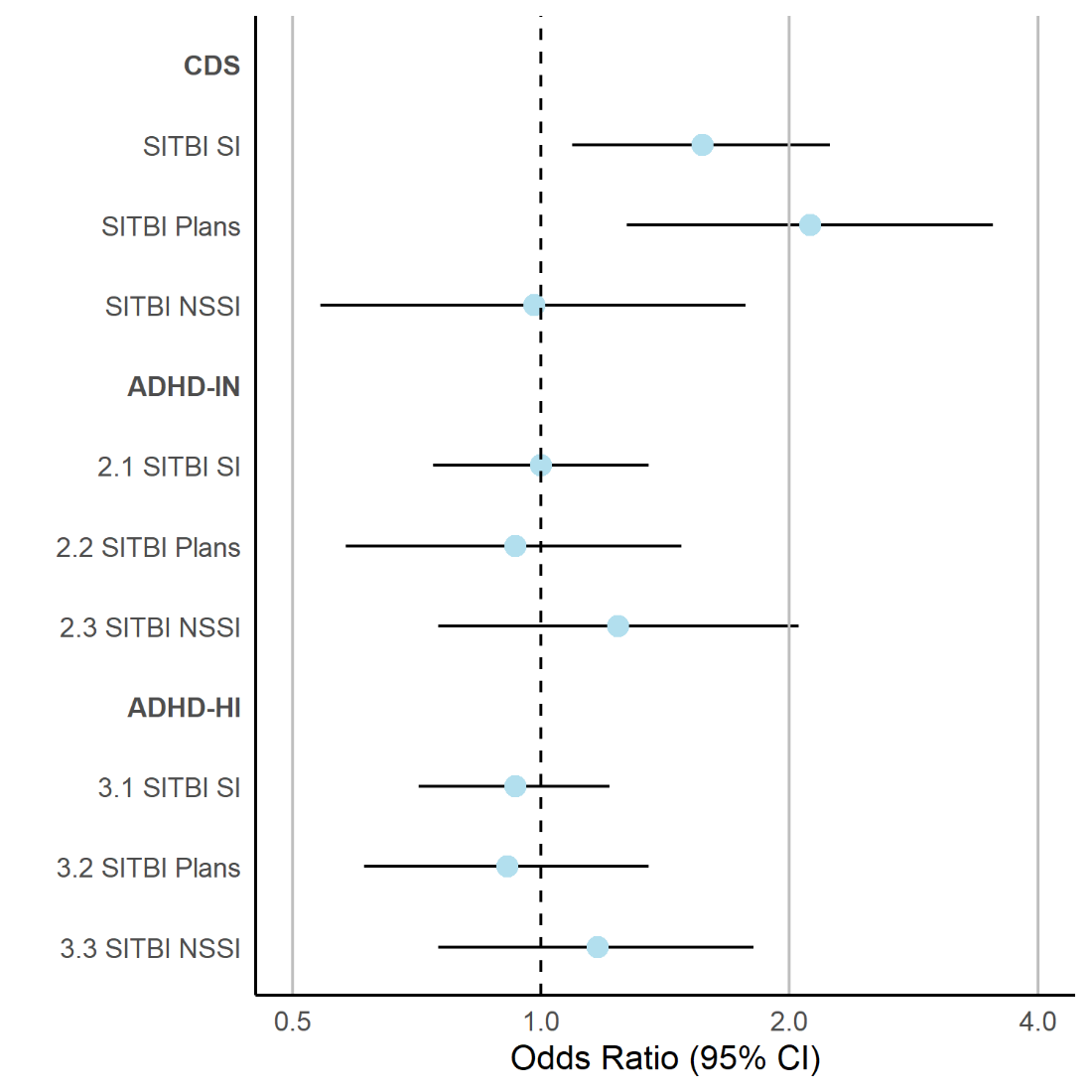

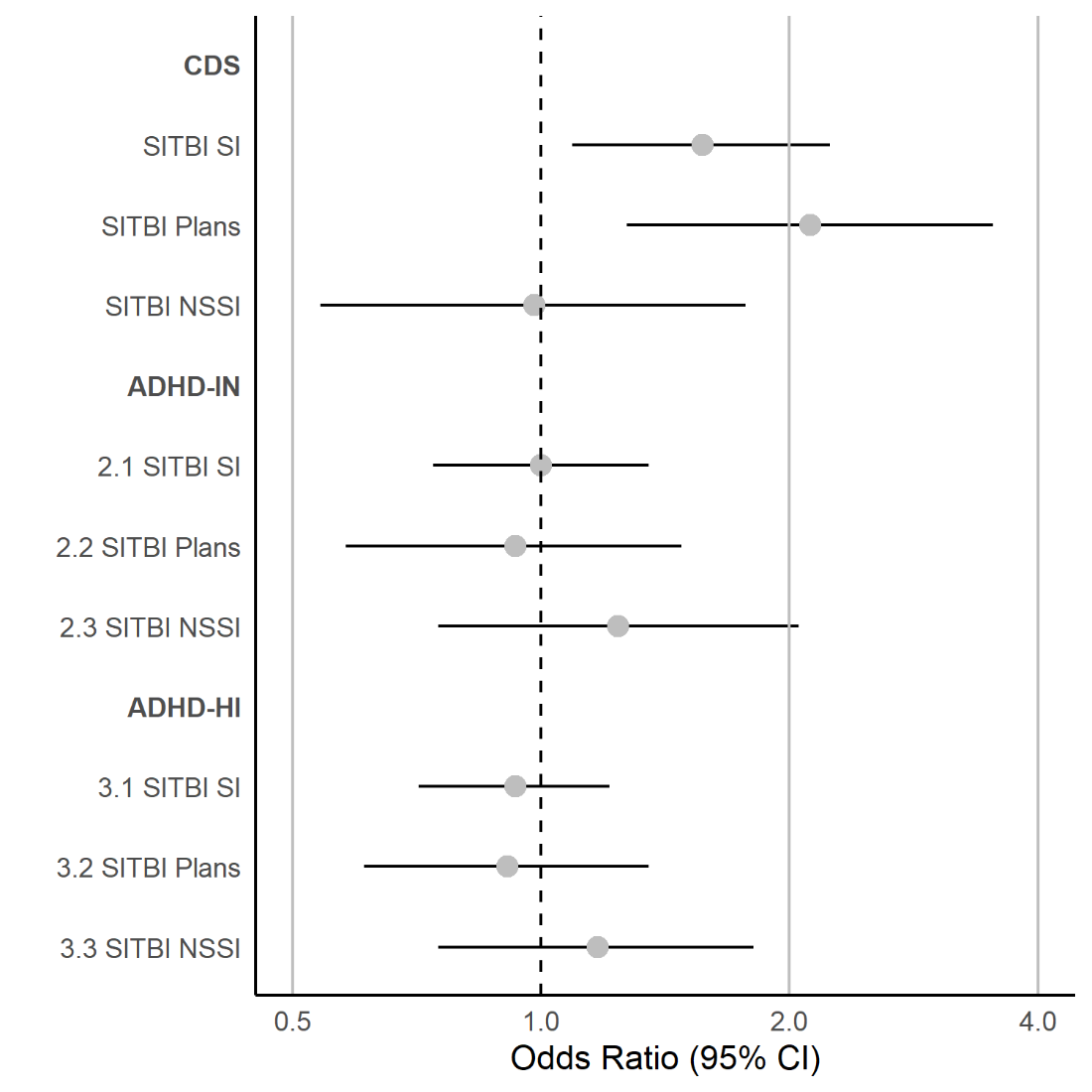

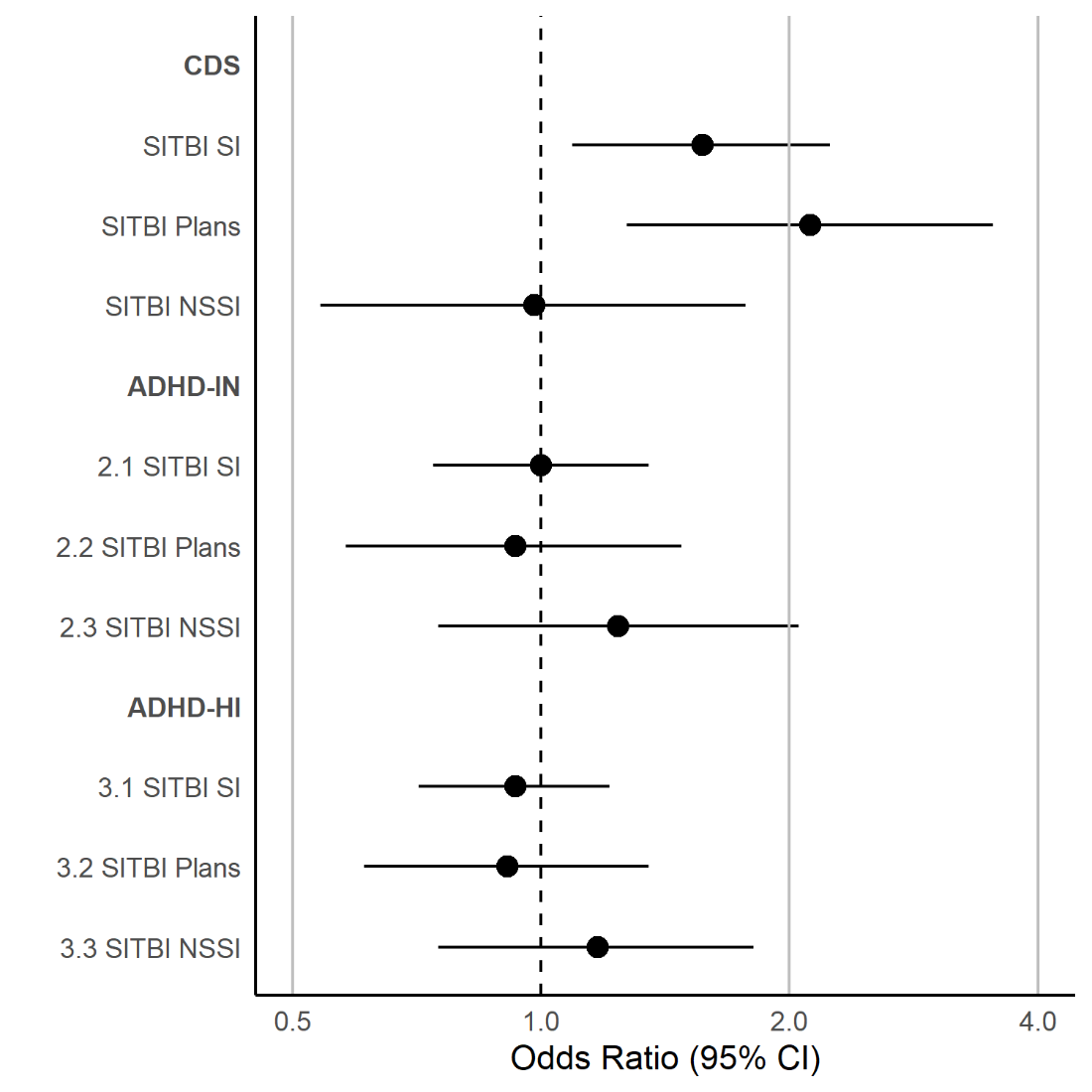


Figure S4

*Logistic analyses examining the unique effects of ADHD-IN, ADHD-HI, and CDS symptoms in relation to SITBI indices associated in models adjusted for demographic characteristics*


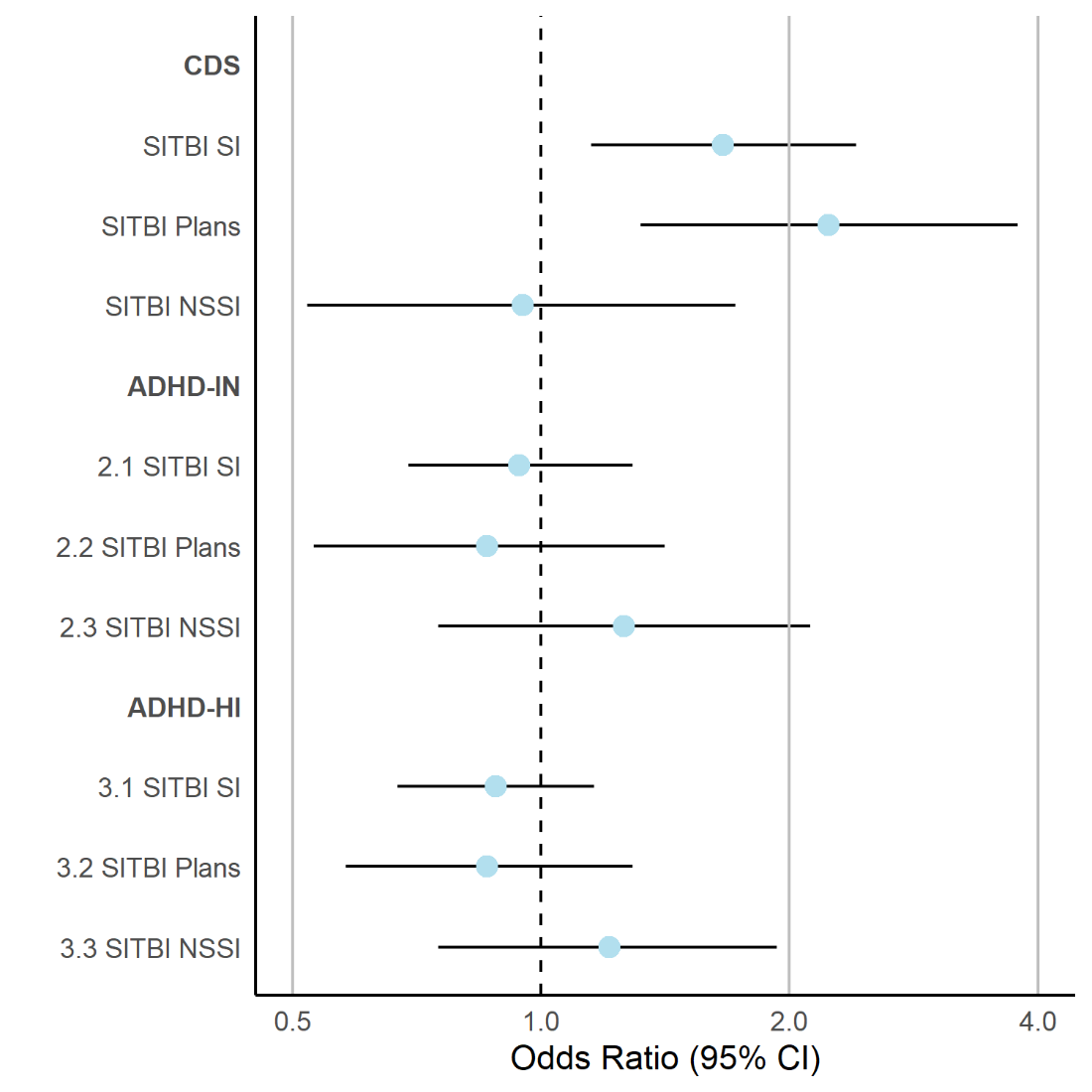

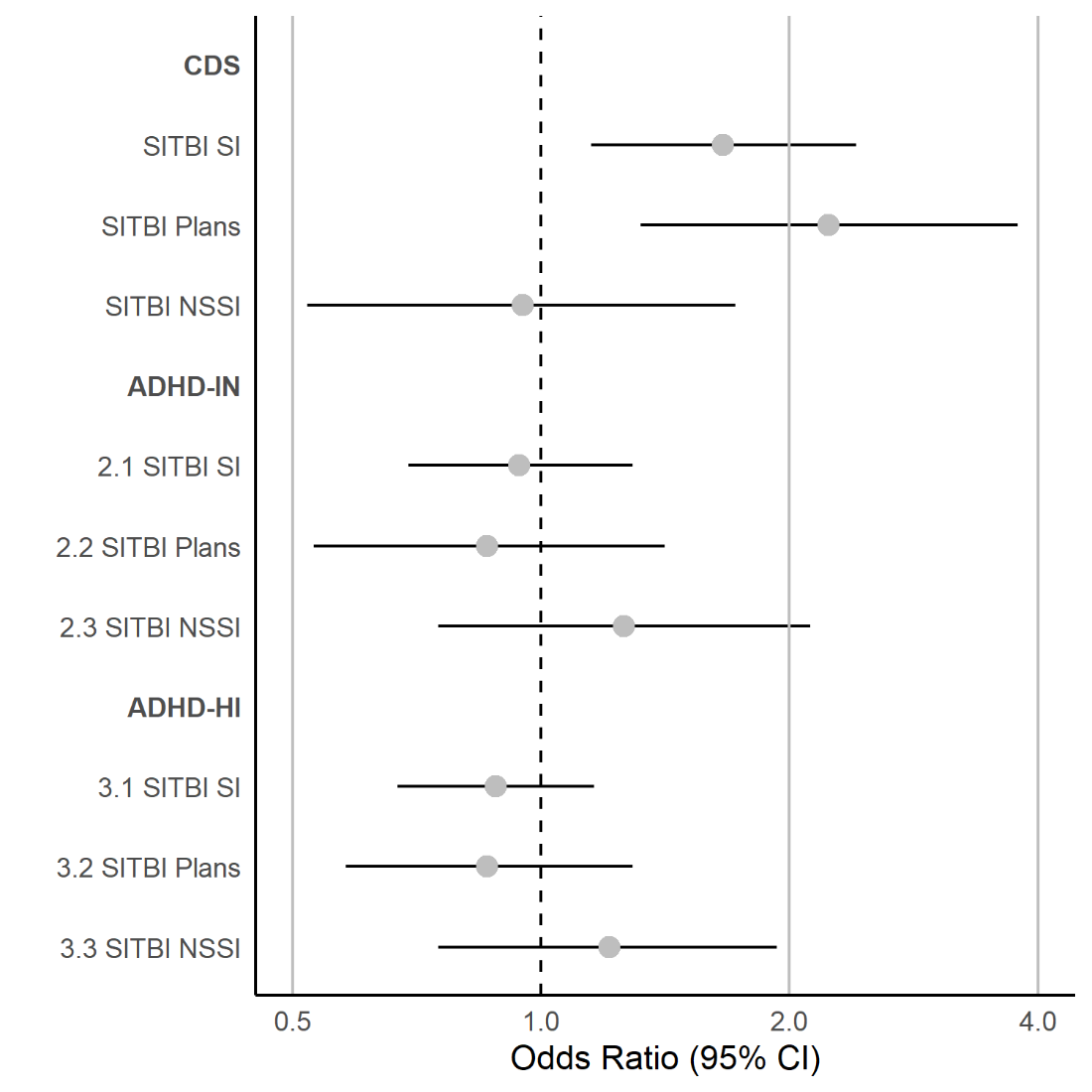

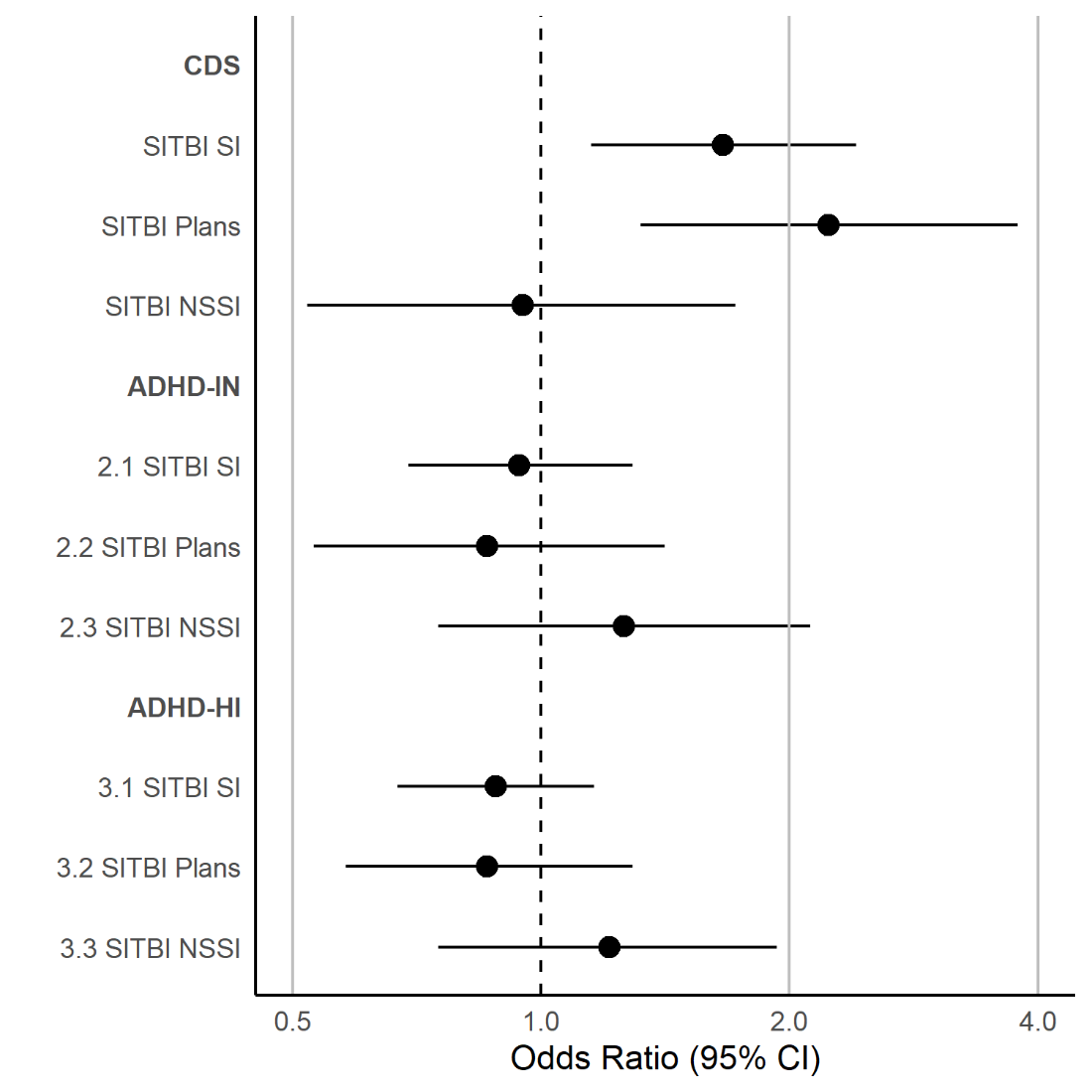

Supplement: Supplementary file 1 — Supplementary Material 1 [file 787_2024_2556_MOESM1_ESM.docx]
